# Supplementary material for: How does climate change affect potential yields of four staple grain crops worldwide by 2030?
Source: PLoS One. 2024 May 31;19(5):e0303857. doi: 10.1371/journal.pone.0303857 (PMC11142697; doi:10.1371/journal.pone.0303857)
Supplement: S1 File — (DOCX) [file pone.0303857.s001.docx]

**Supporting Information**

**Table S1**. Average and top yields (kg/ha) of rice, wheat, maize and soybean worldwide, and global mean temperature (℃) and land precipitation (mm) from 1961 to 2021.

| Year | GMT | PRCP | Rice | | Wheat | | Maize | | Soybean | |
| --- | --- | --- | --- | --- | --- | --- | --- | --- | --- | --- |
|  |  |  | Average | Top | Average | Top | Average | Top | Average | Top |
| 1961 | 14.0 | 886 | 1869 | 6444 | 1089 | 4121 | 1942 | 5000 | 1129 | 2103 |
| 1962 | 13.8 | 861 | 1896 | 6844 | 1206 | 4548 | 1980 | 9419 | 1138 | 2076 |
| 1963 | 13.9 | 885 | 2057 | 6667 | 1132 | 4196 | 2031 | 7000 | 1156 | 2051 |
| 1964 | 13.5 | 865 | 2103 | 6600 | 1241 | 4706 | 1994 | 7917 | 1132 | 3056 |
| 1965 | 13.6 | 839 | 2035 | 6337 | 1215 | 4457 | 2124 | 7917 | 1228 | 2109 |
| 1966 | 14.0 | 871 | 2078 | 7176 | 1408 | 4275 | 2208 | 7400 | 1372 | 2290 |
| 1967 | 13.7 | 853 | 2175 | 7407 | 1339 | 4791 | 2424 | 7333 | 1349 | 2046 |
| 1968 | 13.7 | 869 | 2233 | 7418 | 1453 | 4814 | 2289 | 8500 | 1435 | 3394 |
| 1969 | 13.7 | 872 | 2254 | 7924 | 1417 | 4395 | 2421 | 7167 | 1452 | 2797 |
| 1970 | 13.9 | 885 | 2381 | 6571 | 1494 | 4546 | 2351 | 7247 | 1480 | 2364 |
| 1971 | 13.7 | 880 | 2362 | 7392 | 1625 | 4969 | 2653 | 8444 | 1519 | 2364 |
| 1972 | 13.6 | 845 | 2325 | 6571 | 1605 | 4570 | 2687 | 7850 | 1490 | 2456 |
| 1973 | 14.0 | 896 | 2452 | 6851 | 1684 | 5255 | 2722 | 9139 | 1585 | 2855 |
| 1974 | 13.6 | 889 | 2425 | 6571 | 1616 | 5733 | 2557 | 7055 | 1408 | 2669 |
| 1975 | 13.9 | 897 | 2519 | 7059 | 1570 | 5102 | 2813 | 7666 | 1657 | 3000 |
| 1976 | 13.4 | 863 | 2451 | 7059 | 1791 | 5437 | 2837 | 7087 | 1544 | 3000 |
| 1977 | 14.0 | 867 | 2572 | 7941 | 1672 | 5230 | 2967 | 7375 | 1756 | 3143 |
| 1978 | 13.8 | 880 | 2684 | 7941 | 1933 | 6567 | 3156 | 7143 | 1626 | 3000 |
| 1979 | 14.0 | 857 | 2659 | 8788 | 1852 | 5938 | 3385 | 8032 | 1749 | 3750 |
| 1980 | 14.1 | 861 | 2748 | 8788 | 1855 | 6202 | 3153 | 8076 | 1600 | 4456 |
| 1981 | 14.2 | 865 | 2827 | 9846 | 1880 | 6701 | 3493 | 11000 | 1754 | 2893 |
| 1982 | 13.9 | 841 | 2980 | 9846 | 1999 | 7390 | 3609 | 9071 | 1759 | 3136 |
| 1983 | 14.2 | 838 | 3137 | 9941 | 2126 | 7037 | 2945 | 9156 | 1620 | 4622 |
| 1984 | 13.9 | 858 | 3226 | 8725 | 2220 | 7885 | 3526 | 9359 | 1714 | 3040 |
| 1985 | 13.7 | 839 | 3257 | 7500 | 2172 | 6645 | 3720 | 18100 | 1906 | 3309 |
| 1986 | 14.0 | 849 | 3244 | 7500 | 2321 | 7998 | 3628 | 15494 | 1820 | 3469 |
| 1987 | 14.2 | 817 | 3265 | 7500 | 2290 | 7065 | 3486 | 19229 | 1905 | 4748 |
| 1988 | 14.3 | 896 | 3330 | 7500 | 2293 | 7765 | 3100 | 14778 | 1705 | 4030 |
| 1989 | 14.2 | 889 | 3454 | 8299 | 2373 | 7598 | 3619 | 15000 | 1829 | 5947 |
| 1990 | 14.4 | 861 | 3529 | 9149 | 2563 | 8531 | 3691 | 13794 | 1896 | 3359 |
| 1991 | 14.2 | 843 | 3536 | 8843 | 2444 | 7865 | 3700 | 20430 | 1873 | 3683 |
| 1992 | 14.0 | 822 | 3585 | 8977 | 2540 | 8015 | 3906 | 22450 | 2031 | 3132 |
| 1993 | 14.1 | 849 | 3616 | 8127 | 2544 | 8771 | 3631 | 20020 | 1936 | 3155 |
| 1994 | 14.3 | 846 | 3658 | 10177 | 2448 | 8067 | 4112 | 19024 | 2183 | 3350 |
| 1995 | 14.6 | 871 | 3658 | 9722 | 2515 | 8619 | 3810 | 19048 | 2031 | 3753 |
| 1996 | 14.1 | 884 | 3784 | 8291 | 2577 | 8997 | 4206 | 18711 | 2132 | 3695 |
| 1997 | 14.5 | 852 | 3817 | 8415 | 2702 | 7934 | 4151 | 18667 | 2157 | 3802 |
| 1998 | 14.8 | 912 | 3816 | 9426 | 2706 | 8252 | 4435 | 20453 | 2256 | 3504 |
| 1999 | 14.6 | 919 | 3897 | 9162 | 2751 | 8767 | 4426 | 20659 | 2190 | 3533 |
| 2000 | 14.5 | 928 | 3887 | 9103 | 2722 | 9454 | 4324 | 20867 | 2171 | 3939 |
| 2001 | 14.6 | 898 | 3950 | 9307 | 2742 | 9060 | 4478 | 21100 | 2306 | 3812 |
| 2002 | 14.8 | 874 | 3863 | 9389 | 2755 | 8444 | 4388 | 28675 | 2293 | 3861 |
| 2003 | 14.6 | 881 | 3954 | 10110 | 2652 | 8744 | 4461 | 23099 | 2280 | 3459 |
| 2004 | 14.7 | 896 | 4030 | 9838 | 2943 | 9924 | 4945 | 22115 | 2243 | 3571 |
| 2005 | 14.8 | 862 | 4085 | 9987 | 2829 | 8593 | 4820 | 23315 | 2318 | 3630 |
| 2006 | 14.7 | 910 | 4119 | 10075 | 2891 | 9154 | 4772 | 22514 | 2324 | 3969 |
| 2007 | 14.9 | 911 | 4227 | 9768 | 2815 | 8497 | 4998 | 26827 | 2437 | 3535 |
| 2008 | 14.7 | 892 | 4292 | 9735 | 3062 | 9939 | 5083 | 26830 | 2398 | 3649 |
| 2009 | 14.7 | 866 | 4345 | 9593 | 3037 | 9465 | 5164 | 26825 | 2248 | 3657 |
| 2010 | 14.9 | 923 | 4336 | 10390 | 2971 | 8909 | 5190 | 29236 | 2578 | 3687 |
| 2011 | 14.6 | 922 | 4464 | 9567 | 3164 | 9864 | 5175 | 33816 | 2520 | 3870 |
| 2012 | 14.8 | 887 | 4540 | 9530 | 3089 | 8925 | 4889 | 25879 | 2289 | 3639 |
| 2013 | 14.7 | 913 | 4510 | 10218 | 3250 | 9105 | 5461 | 36762 | 2500 | 4161 |
| 2014 | 14.8 | 862 | 4557 | 10683 | 3315 | 10014 | 5623 | 34098 | 2604 | 4371 |
| 2015 | 15.1 | 826 | 4604 | 9910 | 3317 | 10668 | 5538 | 35291 | 2676 | 4400 |
| 2016 | 15.3 | 858 | 4637 | 10289 | 3405 | 9539 | 5640 | 40413 | 2756 | 4322 |
| 2017 | 15.1 | 888 | 4602 | 9821 | 3541 | 10172 | 5897 | 30802 | 2849 | 4421 |
| 2018 | 15.0 | 855 | 4679 | 10386 | 3425 | 8960 | 5924 | 28466 | 2791 | 4262 |
| 2019 | 15.3 | 827 | 4662 | 8771 | 3547 | 9379 | 5824 | 30167 | 2769 | 4250 |
| 2020 | 15.4 | 939 | 4609 | 10031 | 3474 | 9933 | 5755 | 29650 | 2784 | 4418 |
| 2021 | 15.2 | 931 | 4764 | 10203 | 3492 | 10077 | 5879 | 30314 | 2870 | 4147 |

Note: GMT stands for global mean temperature whereas PRCP for land precipitation of the earth.

**Table S2.** ARIMA-TR model projected average yields (kg/ha) of four staple grain crops worldwide between 2020 and 2030.

| **Crop** | **2020** | **2021** | **2022** | **2023** | **2024** | **2025** | **2026** | **2027** | **2028** | **2029** | **2030** |
| --- | --- | --- | --- | --- | --- | --- | --- | --- | --- | --- | --- |
| Rice | 4793 | 4835 | 4876 | 4917 | 4958 | 4998 | 5038 | 5078 | 5117 | 5156 | 5195 |
| Wheat | 3479 | 3513 | 3548 | 3582 | 3616 | 3650 | 3684 | 3717 | 3751 | 3784 | 3817 |
| Maize | 5937 | 6016 | 6096 | 6176 | 6257 | 6338 | 6419 | 6501 | 6583 | 6665 | 6748 |
| Soybean | 2776 | 2805 | 2834 | 2863 | 2893 | 2922 | 2952 | 2981 | 3011 | 3041 | 3071 |

**Table S3.** Values of *a* and *b* in GS model predicting average yields of four staple grain crops in 2020 and 2021.

| **Year** | **Value** | **Rice** | **Wheat** | **Maize** | **Soybean** |
| --- | --- | --- | --- | --- | --- |
| 2020 | *a* | 0.00842286 | 0.01123333 | 0.00667600 | 0.01394576 |
|  | *b* | 4083.019300 | 4081.155091 | 4080.365820 | 4081.347276 |
| 2021 | *a* | 0.009779472 | 0.013019109 | 0.007769842 | 0.016310485 |
|  | *b* | 4150.488051 | 4149.938914 | 4149.671856 | 4151.100372 |

**Table S4.** ARIMA-TR model projected top yields (kg/ha) of four staple grain crops worldwide between 2020 and 2030.

| **Crop** | **2020** | **2021** | **2022** | **2023** | **2024** | **2025** | **2026** | **2027** | **2028** | **2029** | **2030** |
| --- | --- | --- | --- | --- | --- | --- | --- | --- | --- | --- | --- |
| Rice | 10105 | 10127 | 10148 | 10168 | 10187 | 10204 | 10220 | 10234 | 10247 | 10259 | 10269 |
| Wheat | 9856 | 9886 | 9914 | 9939 | 9961 | 9981 | 9998 | 10013 | 10025 | 10035 | 10042 |
| Maize | 36711 | 37589 | 38479 | 39381 | 40294 | 41220 | 42157 | 43105 | 44066 | 45038 | 46022 |
| Soybean | 4104 | 4118 | 4132 | 4146 | 4159 | 4173 | 4186 | 4199 | 4212 | 4224 | 4237 |

**Table S5.** Values of *a* and *b* in GS model predicting top yields of four staple grain crops in 2020 and 2021.

| **Year** | **Value** | **Rice** | **Wheat** | **Maize** | **Soybean** |
| --- | --- | --- | --- | --- | --- |
| 2020 | *a* | 0.00393044 | 0.00409383 | 0.00124238 | 0.00903458 |
|  | *b* | 4084.231821 | 4088.574962 | 4095.793201 | 4084.271961 |
| 2021 | *a* | 0.004713669 | 0.004820576 | 0.001536707 | 0.010568091 |
|  | *b* | 4155.447736 | 4153.255287 | 4168.370144 | 4151.816600 |

**Table S6.** Ratios (%) of average to top yields of four staple grain crops worldwide between 2020 and 2030.

| **Crop** | **2020** | **2021** | **2022** | **2023** | **2024** | **2025** | **2026** | **2027** | **2028** | **2029** | **2030** |
| --- | --- | --- | --- | --- | --- | --- | --- | --- | --- | --- | --- |
| Rice | 47.4 | 47.7 | 48.1 | 48.4 | 48.7 | 49.0 | 49.3 | 49.6 | 49.9 | 50.3 | 50.6 |
| Wheat | 35.3 | 35.5 | 35.8 | 36.0 | 36.3 | 36.6 | 36.9 | 37.1 | 37.4 | 37.7 | 38.0 |
| Maize | 16.2 | 16.0 | 15.8 | 15.7 | 15.5 | 15.4 | 15.2 | 15.1 | 14.9 | 14.8 | 14.7 |
| Soybean | 67.6 | 68.1 | 68.6 | 69.1 | 69.6 | 70.0 | 70.5 | 71.0 | 71.5 | 72.0 | 72.5 |

**Table S7.** Model results of global warming effect on average and top yields of four staple grain crops from 1961 to 2021 and to 2030.

| **Crop** | **Yield** | **Equation** | **Model Summary and Parameter Estimates** | | | | | |
| --- | --- | --- | --- | --- | --- | --- | --- | --- |
|  |  |  | R Square | F | Constant | b1 | b2 | b3 |
| Rice | Average | Cubic | 0.881 | 248.089 | -49375.212 | 4726.317 | 0.000 | -5.052 |
|  | Top | Cubic | 0.735 | 93.084 | -144849.215 | 15012.653 | 0.000 | -20.851 |
| Wheat | Average | Inverse | 0.874 | 470.669 | 20226.683 | -255498.394 |  |  |
|  | Top | Cubic | 0.783 | 82.978 | -206195.846 | 20731.205 | 0.000 | -28.221 |
| Maize | Average | Linear | 0.893 | 565.664 | -26827.145 | 2144.709 |  |  |
|  | Top | Quadratic | 0.888 | 266.134 | 506553.029 | -85290.472 | 3570.296 |  |
| Soybean | Average | Linear | 0.897 | 590.360 | -10277.017 | 855.611 |  |  |
|  | Top | Quadratic | 0.478 | 30.703 | -94521.524 | 12704.740 | -408.789 |  |

Note: all equations are at great significance level of 1.0%.

**Table S8.** Model results of global rainfall effect on average and top yields of four staple grain crops from 1961 to 2021 and to 2030.

| **Crop** | **Yield** | **Equation** | **Sig.** | **Model Summary and Parameter Estimates** | | | | | |
| --- | --- | --- | --- | --- | --- | --- | --- | --- | --- |
|  |  |  |  | R Square | F | Constant | b1 | b2 | b3 |
| Rice | Average | Quadratic | 0.001 | 0.256 | 11.503 | 254700.392 | -586.986 | 0.342 |  |
|  | Top | Quadratic | 0.005 | 0.145 | 5.693 | 237041.827 | -535.306 | 0.313 |  |
| Wheat | Average | Quadratic | 0.000 | 0.241 | 10.627 | 204068.981 | -470.599 | 0.274 |  |
|  | Top | Quadratic | 0.000 | 0.218 | 9.322 | 518366.755 | -1189.566 | 0.692 |  |
| Maize | Average | Quadratic | 0.000 | 0.253 | 11.359 | 330503.014 | -764.919 | 0.447 |  |
|  | Top | Quadratic | 0.000 | 0.267 | 12.189 | 3102198.763 | -7208.612 | 4.209 |  |
| Soybean | Average | Quadratic | 0.000 | 0.261 | 11.827 | 139342.736 | -321.228 | 0.188 |  |
|  | Top | Quadratic | 0.017 | 0.115 | 4.355 | 181990.680 | -411.977 | 0.238 |  |
